# P05-02 Implementing exercise based injury prevention programs -lessons learned

**DOI:** 10.1093/eurpub/ckac095.069

**Published:** 2022-08-29

**Authors:** Joske Nauta

**Affiliations:** Public and Occupational Health, Amsterdam University Medical Center, Amsterdam, The Netherlands

**Keywords:** Injury prevention, physical activity promotion

## Abstract

**Background:**

In youth, physical activity is for a large part accumulated through participating in sports clubs and gymnastic classes. One of the reasons youth drop-out from sports and physical education participation are the injuries they sustain while being active. And, although we know that exercise based injury prevention programs can reduce the physical activity related injury risk, it has proven to be difficult to convince coaches, trainers and physical education teachers to implement these preventive exercises in their training/teaching routines. This presentation will focus on the lessons learned regarding the experiences and views of coaches, trainers and physical education teachers that participated in our injury prevention trials.

**Methods:**

Over the past decade, we have evaluated the effectiveness of several physical activity based injury prevention programs that were used in both the sports as well as the school setting. Alongside these effectiveness trails, process evaluations have been conducted to assess if the interventions were delivered and received as intended and what their views were regarding implementation of the exercises in their daily routine.

**Results:**

A comparison will be made between views of coaches, trainers and physical education teachers that participated in our several trials.

**Conclusions:**

Combining the views of coaches, trainers and physical education teachers regarding future implementation of injury prevention programs will help guide the implementation of the exercised based preventive routines that will be developed for the EU funded Move Healthy project.

